# Identification of ferroptosis-related genes in male mice with sepsis-induced acute lung injury based on transcriptome sequencing

**DOI:** 10.1186/s12890-023-02361-3

**Published:** 2023-04-20

**Authors:** Wen Hu, Zhen Wu, Mei Zhang, Shilin Yu, Xiaohua Zou

**Affiliations:** 1 Guizhou Medical University, Guiyang, 550004 Guizhou China; 2 The Affiliated Hospital of Guizhou Medical University, Guiyang, 550004 Guizhou China

**Keywords:** Transcriptome sequencing, Ferroptosis, Gene set enrichment analysis, Machine learning methods

## Abstract

**Background:**

Sepsis can result in acute lung injury (ALI). Studies have shown that pharmacological inhibition of ferroptosis can treat ALI. However, the regulatory mechanisms of ferroptosis in sepsis-induced ALI remain unclear.

**Methods:**

Transcriptome sequencing was performed on lung tissue samples from 10 sepsis-induced mouse models of ALI and 10 control mice. After quality control measures, clean data were used to screen for differentially expressed genes (DEGs) between the groups. The DEGs were then overlapped with ferroptosis-related genes (FRGs) to obtain ferroptosis-related DEGs (FR-DEGs). Subsequently, least absolute shrinkage and selection operator (Lasso) and Support Vector Machine-Recursive Feature Elimination (SVM-RFE) were used to obtain key genes. In addition, Ingenuity Pathway Analysis (IPA) was employed to explore the disease, function, and canonical pathways related to the key genes. Gene set enrichment analysis (GSEA) was used to investigate the functions of the key genes, and regulatory miRNAs of key genes were predicted using the NetworkAnalyst and StarBase databases. Finally, the expression of key genes was validated with the GSE165226 and GSE168796 datasets sourced from the Gene Expression Omnibus (GEO) database and using quantitative real-time polymerase chain reaction (qRT-PCR).

**Results:**

Thirty-three FR-DEGs were identified between 1843 DEGs and 259 FRGs. Three key genes, *Ncf2*, *Steap3*, and *Gclc*, were identified based on diagnostic models established by the two machine learning methods. They are mainly involved in infection, immunity, and apoptosis, including lymphatic system cell migration and lymphocyte and T cell responses. Additionally, the GSEA suggested that *Ncf2* and *Steap3* were similarly enriched in mRNA processing, response to peptides, and leukocyte differentiation. Furthermore, a key gene-miRNA network including 2 key genes (*Steap3* and *Gclc*) and 122 miRNAs, and a gene-miRNA network with 1 key gene (*Steap3*) and 3 miRNAs were constructed using NetworkAnalyst and StarBase, respectively. Both databases predicted that mmu-miR-15a-5p was the target miRNA of *Steap3*. Finally, *Ncf2* expression was validated using both datasets and qRT-PCR, and *Steap3* was validated using GSE165226 and qRT-PCR.

**Conclusions:**

This study identified two FR-DEGs (*Ncf2* and *Steap3*) associated with sepsis-induced ALI via transcriptome analyses, as well as their functional and metabolic pathways.

**Supplementary Information:**

The online version contains supplementary material available at 10.1186/s12890-023-02361-3.

## Background

Sepsis is a serious disease caused by inflammation and systemic infection [1]. Over 40% of patients with sepsis experience acute lung injury (ALI), characterized by neutrophilic inflammation and pulmonary vascular hyperpermeability [2]. Sepsis causes inflammation in the lungs, which is difficult to resolve and causes diffuse interstitial or alveolar edema, decreased lung compliance, and hypoxemia [3]. Thus, ALI can result in irreversible damage to the lungs [4]. Sepsis-induced ALI has a higher mortality rate than ALI caused by other factors [5]. Currently, sepsis treatment is not effective in preventing septic ALI because its pathogenesis remains unclear, and further research in this field is urgently needed.

Ferroptosis is a recently discovered pattern of programmed cell death that differs from iron-dependent apoptosis, necrosis, and autophagy. A unique characteristic of ferroptosis is lipid peroxidation during intracellular iron accumulation, leading to cell death [6]. Recent studies have found that ferroptosis can exacerbate sepsis-induced injuries to many organs, such as in heart [7], acute lung [8, 9], and acute kidney injuries [10]. Researchers have found that ferroptosis inhibitors (Ferrostatin-1) can alleviate ferroptosis in human bronchial epithelial cells (BEAS-2B) by downregulating the expression of intracellular ferroptosis markers SLC7A11 and GPX4 and significantly increasing malondialdehyde (MDA) and total iron levels, which can promote cell survival [9]. Additionally, it has been reported that itaconate, a macrophage metabolic reprogramming product, inhibits macrophage ferroptosis by activating the Nrf2 pathway [11]. LPS-induced ALI is significantly influenced by ferroptosis, as indicated in the above studies. Therefore, the search for ferroptosis-related genes in sepsis-induced ALI is crucial for the clinical diagnosis of patients, and could contribute to the development of novel early interventions.

To date, there have been no bioinformatic studies of ferroptosis-related genes for sepsis-induced ALI. Therefore, this study aimed to analyze sequencing data to identify disease-related ferroptosis genes and their mechanisms by sequencing mRNA in mouse models of septic lung injury and normal mice, and performing bioinformatics analysis. We aimed to provide a novel reference for the diagnosis and treatment of septic lung injury.

## Methods

### Modeling and sampling of animals

Twenty 6 to 8-week-old C57 male mice (18–20 g; Beijing Huafukang Biotechnology Company, Beijing, China) were raised and housed in a pathogen-free environment with adequate water and food. Mice were randomly divided into the model and control groups (*n = *10 per group). Mouse sepsis models were established using cecal ligation and puncture (CLP) as previously described [12], according to the observation, the activity, eating and drinking of mice in the model group were significantly reduced 12 h after operation, and the mortality rate was about 40% after 24 h. Briefly, mice were fixed on an operating table after anesthesia. The middle and lower abdominal hair were scraped using a razor. The cecum was obtained by making a 1 cm incision in the middle of the abdomen along the ventral white line, and it was gently kneaded with forceps after sterilization. One-third of the cecum was ligated from the bottom of the abdominal cavity using 3–0 sterile silk. The cecum was placed back into the abdominal cavity once the intestinal contents were squeezed out twice with an 18-G needle. Layers of 4–0 mycelium thread were used for suturing and the area was sterilized again. Sham mice underwent the same procedure without puncturing or ligation. Left lung tissues were obtained after 24 h. To ensure that the samples were suitable for later analysis, they were stored at − 80 ℃ or fixed in 4% paraformaldehyde.

### Observation of lung morphology and lung injury assessment using H&E staining

The left lung tissues were embedded in paraffin and cut into 4-μm thick sections. H&E staining was performed following dehydration and deparaffinization of the samples. The slides were examined under a light microscope (Nikon Eclipse Ci-L, Tokyo, Japan) at magnifications of 200 × and 400 × and images were captured. A scoring system was used to evaluate lung injury as previously described [13]

### Upstream data processing and data set acquisition

RNA was extracted from the lung tissue samples of twenty mice (10 sepsis-induced ALI samples and 10 controls), and was further isolated and purified using TRIzol reagent (Invitrogen, Carlsbad, CA, USA) following the manufacturer's procedure. The amount of RNA and purity of each sample were quantified using a NanoDrop ND-1000 (NanoDrop, Wilmington, DE, USA). Subsequently, a Bioanalyzer 2100 (Agilent, CA, USA) with RIN number > 7.0 was used to assess RNA integrity, which was further confirmed using electrophoresis on a denaturing agarose gel. A cDNA library with an insert size of 300 ± 50 bp was constructed according to the manufacturer's instructions. Finally, 2 × 150-bp paired-end sequencing (PE150) was conducted on Illumina Novaseq 6000 (LC-Bio Technology Co., Ltd., Hangzhou, China) according to the vendor’s recommended protocol.

FastQC (version 0.11.9) was used to remove reads with adaptor contamination, low-quality bases, or undetermined bases using the default parameters. Sequence quality was also verified using FastQC and preprocessed using Trimmomatic (version 0.39) to obtain clean data. HISAT2 (https://ccb.jhu.edu/software/hisat2) was used to map reads to the reference genome (GRCm39). Finally, StringTie (https://ccb.jhu.edu/software/stringtie) was used to assemble the reads and estimate the expression level of mRNAs by calculating the FPKM as follows:

$$FPKM = \frac{{total\;ex\;on\;fragments}}{{mapped\;reads\left( {millions} \right) \times ex\;on\;length\left( {kB} \right)}}$$

In addition, the GSE165226 and GSE168796 datasets were downloaded from the GEO database (https://www.ncbi.nlm.nih.gov/geo/). GSE165226 contains the mRNA sequencing data of lung tissue samples from six septic ALI mice and six control mice, and GSE165226 includes twelve post-mortem perfused lung tissue samples from six male CLP sepsis survivor mice and six unoperated healthy controls. A total of 259 ferroptosis-related genes (FRGs) were acquired from the FerrDb and GeneCards databases.

### Screening and functional enrichment of DEGs

To evaluate the reproducibility of overall gene expression in the case and control groups, and whether there were differences in expression between groups, a correlation heat map between the samples was plotted based on the gene expression profile of each sample (Additional file 8: Table S3A). Subsequently, principal component analysis (PCA) was performed on the sepsis-induced ALI and control samples to assess whether the biological replicates of each sample were consistent. DEGs between the 10 sepsis-induced ALI and 10 control samples were screened using the DESeq2 R package (version 1.26.0) with the following thresholds: |log_2_(fold change)|≥ 1, *p* < 0.05, and baseMean ≥ 50. GO and KEGG enrichment analyses were performed on the DEGs using clusterProfiler (version 3.14.3) [14–16].

### Identification of FR-DEGs

To further identify FRGs associated with sepsis-induced ALI, overlap analysis was performed on the DEGs and 259 FRGs. Then, t-tests and ggpubr were used to plot the expression boxplot of the intersected genes in the sepsis-induced ALI and control groups, and genes with significant expression differences were deemed to be FR-DEGs. Furthermore, a protein–protein interaction (PPI) network of FR-DEGs was constructed using the STRING website to detect interactions among them.

### Screening key genes using shrinkage and selection operator (Lasso) and support vector machine-recursive feature elimination (SVM-RFE)

Firstly, LASSO regression analysis was performed on the FR-DEGs using the glmnet R package with five-fold cross validation and the following settings: family = “class” and nlambda = 100. Then, a boxplot was drawn by combining the probabilities of samples in the sepsis-induced ALI group (1) and the control group (0) to determine the predictive ability of the corresponding model for sepsis-induced ALI under lambda.min and lambda.1se, and the ROC curves of the two models were drawn. Subsequently, SVM-RFE regression analysis was performed on the FR-DEGs using e1071 with tenfold cross validation, and the number of features was determined using the lowest 10 × CV error. Finally, the diagnostic genes from LASSO and SVM-RFE were overlapped, and the intersecting genes were deemed as key genes. ROC curves were plotted for the diagnostic genes to evaluate their diagnostic efficiency among the training set and the validation set (GSE165226 and GSE168796).

### Ingenuity pathway analysis (IPA) and gene set enrichment analysis (GSEA)

The expression matrices of key genes and FR-DEGs were used as input data for IPA, including canonical pathway, disease, and function analyses, and the screening thresholds of canonical pathway analysis were set as |zscore|> 2 and *p* < 0.05.

In addition, this study utilized homologous gene conversion to convert the sample matrix expression of homologous genes in mice into human genes. Combining the expression of all the genes in the matrix, GSEA was performed on key genes to explore the potential biological processes and pathways by which key genes are involved in sepsis-induced ALI.

### miRNAs of key genes

Key genes were used as input data to predict interacting miRNAs using NetworkAnalyst (https://www.networkanalyst.ca/) with the miRTarBase and StarBase databases.

### Expression validation of key genes

To ensure the reliability of the expression results of the key genes, expression was verified using t-test in two external validation sets: GSE165226 and GSE168796. In addition, qRT-PCR was performed on seventeen frozen mouse lung samples (eight ALI samples and nine controls).

Total RNA for qRT-PCR was extracted using TRIZol (Thermo Fisher, Shanghai, CN), and reverse-transcribed into cDNA for qPCR reactions using the SureScript-First-strand-cDNA-synthesis-kit (Servicebio, Wuhan, CN). The qRT-PCR reaction consisted of 3 µL cDNA, 5 µL 2xUniversal Blue SYBR Green qPCR Master Mix (Servicebio), and 1 µL each of the forward and reverse primers. PCR was performed in a BIO-RAD CFX96 Touch™ PCR detection system (Bio-Rad Laboratories, Inc., Hercules, CA, USA) under the following thermal cycling conditions: 40 cycles at 95 °C for 60 s, 95 °C for 20 s, 55 °C for 20 s, and 72 °C for 30 s. Gene expression was determined using the 2-△△Ct method, and GraphPad Prism 5 was used to plot and determine statistically significant differences. The housekeeping gene GAPDH was conducted as an internal reference for the key genes. The primers for the key genes are displayed in Additional files 3–6: Tables 1 and 2.

### Statistical analysis

All analyses were conducted using R language (https://www.r-project.org/). The t-test was utilized to contrast the expression differences of the FR-DEGs and key genes between sepsis-induced ALI and control samples. If not specified above, *p* < 0.05 was regarded as statistically significant. 

## Results

### Septic mouse model

**Fig. 1:**
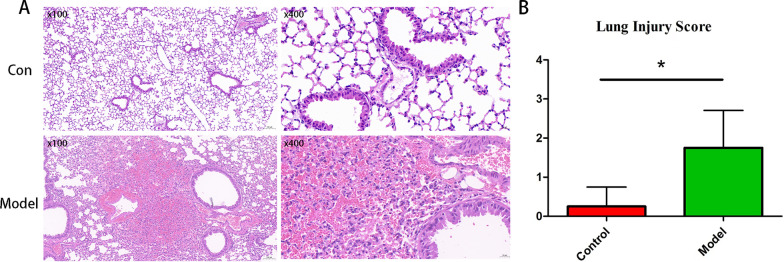
Hematoxylin and eosin (HE) staining and acute lung injury scores of lung samples. **a** Representative HE-stained images of lung tissue. Scale bar, 100 × magnification in the left panels and 400 × magnification in the right panels. **b** The lung injury scores were calculated based on the HE staining; *P < 0.05

Hematoxylin and eosin (HE) staining and light microscopy analyses of lung tissues were conducted 24 h after cecal ligation and puncture (Fig. 1a). The lung tissues of septic mice showed comprehensive morphological changes. Lobar hemorrhage in the lung tissue, a large number of red blood cells in the alveolar space, and some neutrophil infiltration were observed, indicating lung injury. However, no significant lung tissue damage was observed in the sham-operated group. Additionally, the ALI group had a higher lung injury score than that of the sham group (Fig. 1b).

### Preprocessing of raw sequencing data

The raw data are presented in Additional file 3: Table S1A, and the quality control results indicated that the sequence data were of high quality (Additional file 1: Figure S1a, Additional file 2: S1b). The clean data acquired from preprocessing with Trimmomatic are shown in Additional file 4: Table S1B. The base quality of each sequencing fragment was higher than QC30, that is, the base error rate was less than 1/1000, and the number of filtered retained reads was greater than 10 M, indicating that the sequencing depth was greater than 100 × , meeting the requirements of transcriptome analysis. Additionally, comparison between clean data and GRCm39 suggested that the properly mapped rates of the twenty sequencing samples were higher than 90%, indicating that the properly paired rate was high and unaffected by exogenous RNA molecules, making it adequate for subsequent analyses (Additional file 5: Table S1C).

### Functional annotation of differentially expressed genes (DEGs)

**Fig. 2:**
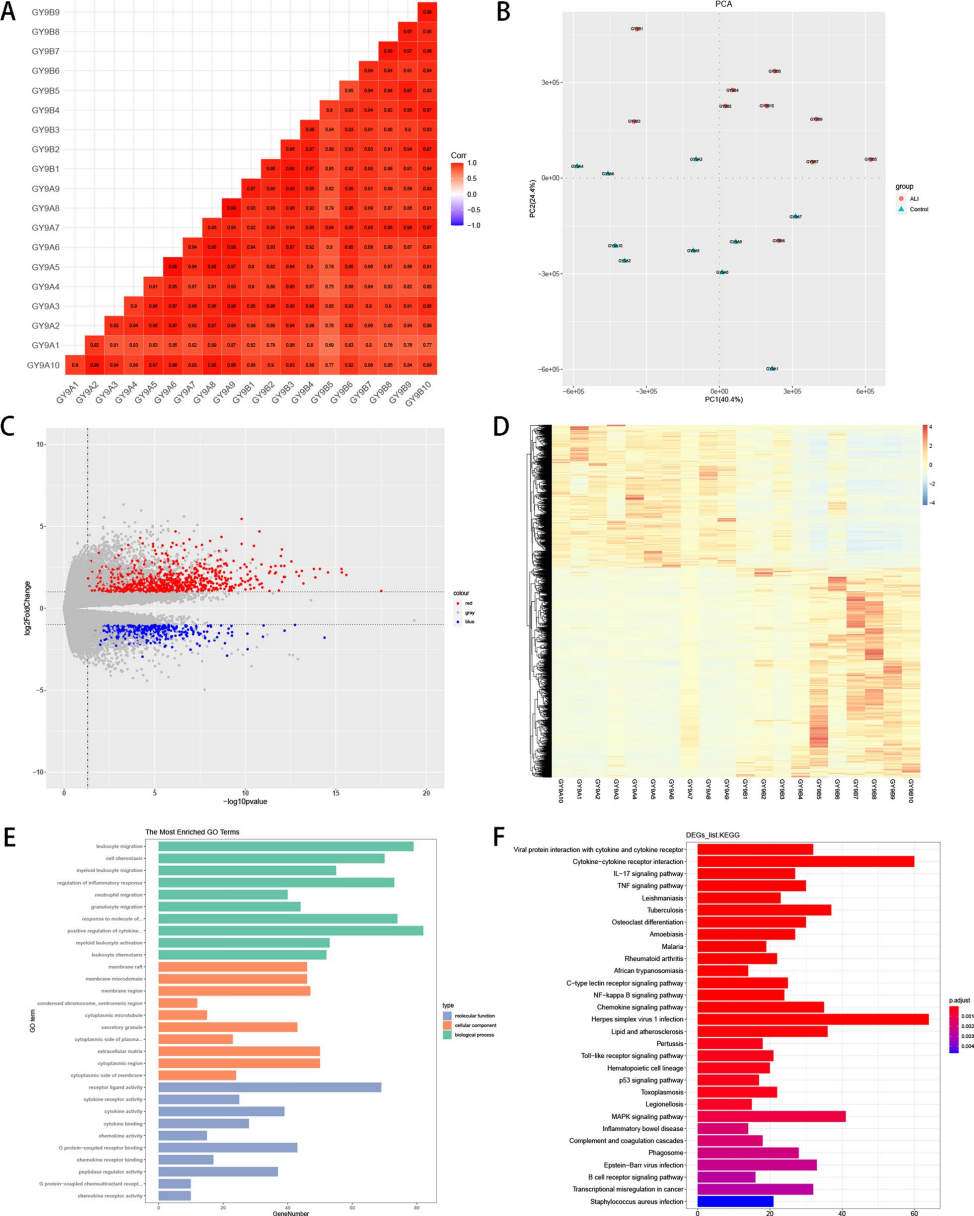
Identification and functional analysis of differentially expressed genes (DEGs). **a** Correlation heat map between twenty samples. **b** Principal Component Analysis. **c** Volcano Plot of DEGs between sepsis-induced ALI and control samples. **d** Heatmap of DEGs. **e**–**f** GO and KEGG bar chart

To evaluate the reproducibility of the overall gene expression of the samples, a correlation heat map was plotted between each of the twenty samples. This revealed that the correlations were adequate and positive, and there were no outlier samples (Fig. 2a). Moreover, the PCA results showed clear boundaries between the case and control samples, and samples within each group were significantly clustered, indicating good sample repetition in each group (Fig. 2b).

One thousand eight hundred and forty-three DEGs were identified between the 10 sepsis-induced ALI and 10 control samples, which included 1135 upregulated and 708 downregulated genes (Fig. 2c, 2d). The DEGs were involved in biological processes (BP) including immune cell migration and responses, such as leukocyte migration, myeloid leukocyte migration, regulation of inflammatory response, granulocyte migration, and neutrophil migration. The molecular functions (MF) mainly involved cytokine activation, including cytokine receptor activity, cytokine activity, chemokine activity, and receptor ligand activity (Fig. 2e). The enriched KEGG pathways were mainly related to cytokine interactions, immune-related signaling pathways, and *Staphylococcus aureus* infection, which may be closely related to sepsis-induced lung injury (Fig. 2f).

### Identification of 26 FR-DEGs

**Fig. 3:**
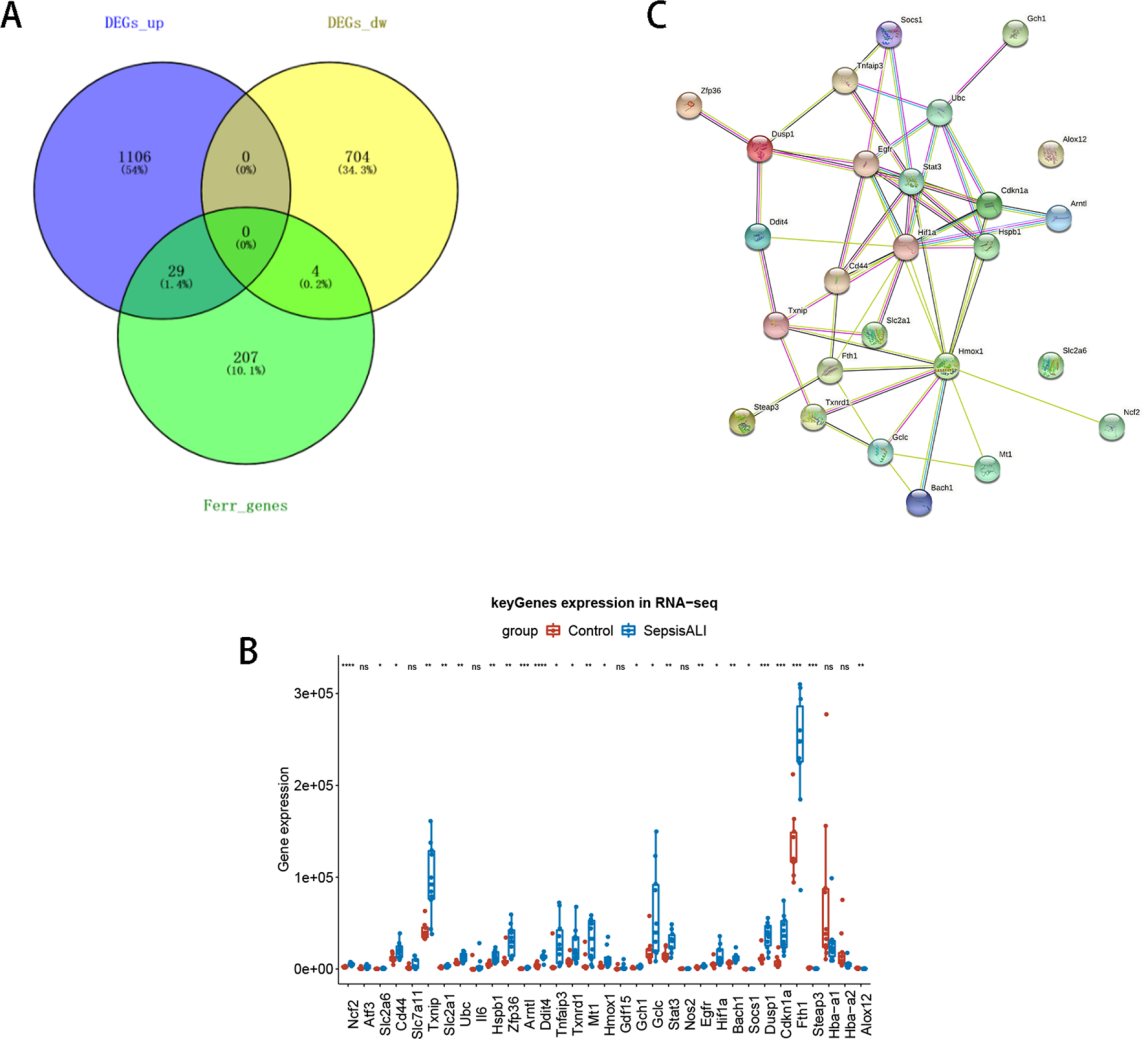
Identification of ferroptosis-related differentially expressed genes (FR-DEGs). **a** Venn diagram of all DEGs and FR genes. **b** Boxplot of intersected gene expressions. **c** Protein–protein interaction network of FR-DEGs

Overlap analysis of the 1843 DEGs and 259 FRGs found 33 intersecting genes (29 upregulated and 4 downregulated) (Fig. 3a). The boxplot revealed 26 differentially expressed genes between the sepsis-induced ALI and control samples: *Ncf2, Slc2a6, Cd44, Txnip, Slc2a1, Ubc, Hspb1, Zfp36, Arntl, Ddit4, Tnfaip3, Txnrd1, Mt1, Hmox1, Gch1, Gclc, Stat3, Egfr, Hif1a, Bach1, Socs1, Dusp1, Cdkn1a, Fth1, Steap3,* and *Alox12* (Fig. 3b). Therefore, these genes were used as FR-DEGs for the construction of a PPI network, which eventually consisted of 24 FR-DEGs (*Alox12* and *Slc2a6* were solitary nodes) (Fig. 3c).

### Identification of three diagnostic genes

**Fig. 4:**
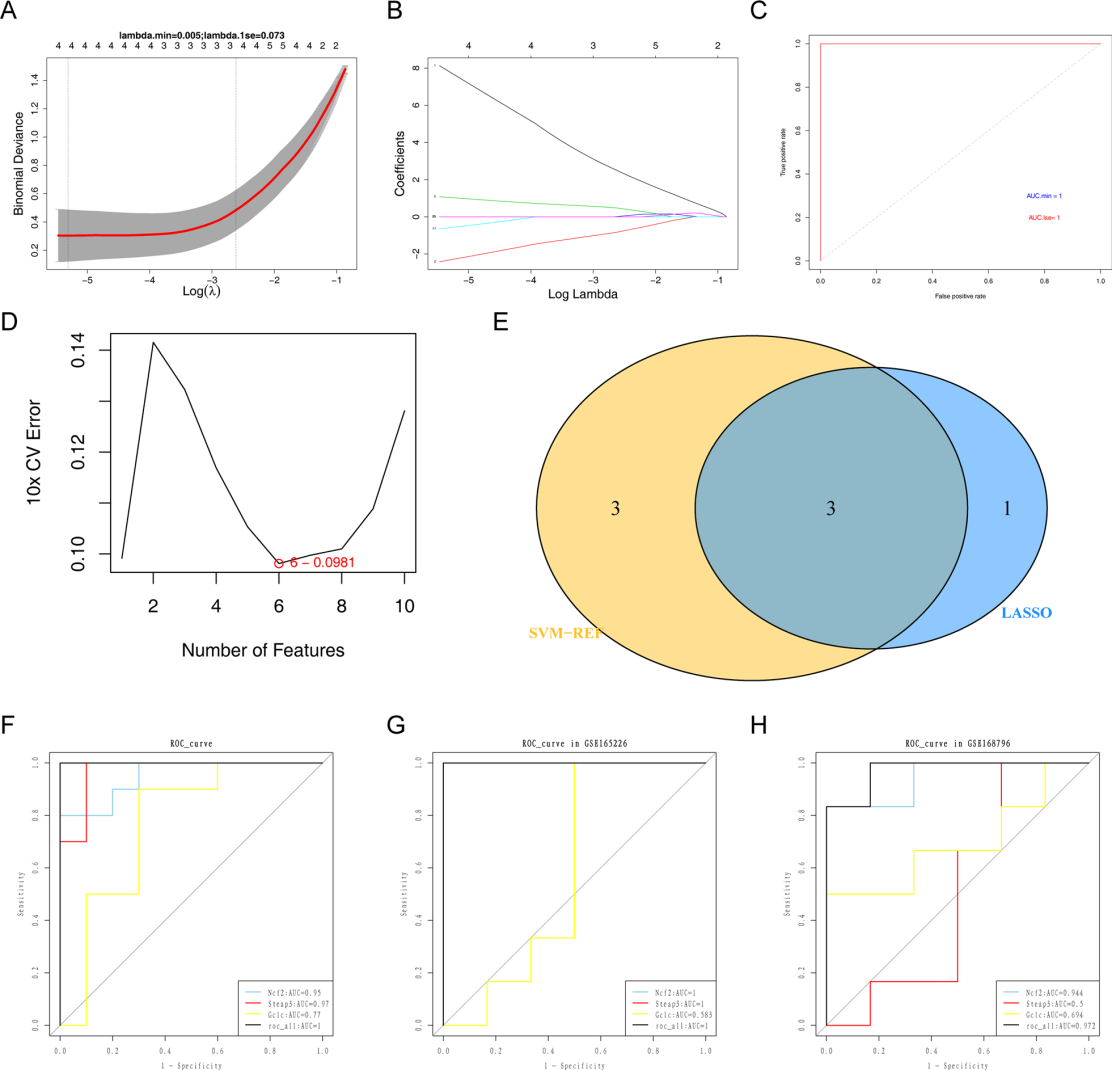
Screening of key genes using LASSO and SVM-RFE. **a** Cross-validation error trend with lambda in LASSO regression and correlation coefficient versus lambda for LASSO regression. **b** lambda. min and lambda.1se model boxplots. **c** ROC curve for the diagnostic model. **d** SVM-RFE error rate curve. **e** Venn diagram of genes in LASSO and SVM-RFE models. **f**–**h** ROC curves for each common gene and the combination in the training set, GSE165226 and GSE168796 cohorts

LASSO analysis of the 26 FR-DEGs revealed a lambda-min model, and lambda.1se models were built for when lambda.min and lambda.1se equaled 0.005 and 0.073, respectively (Fig. 4a). Additionally, the probability minimum model boxplots of the two models showed that the probability of sepsis-induced ALI in the two models was significantly higher than that in the control group. The predictions were consistent with actual values, indicating that the model predictions were accurate (Fig. 4b). The AUCs of the two models were greater than 0.9, indicating that they had good diagnostic ability (Fig. 4c). Considering the detection accuracy of the model in clinical applications, the LASSO model using lambda.min was optimal, and four diagnostic model genes were obtained: *Ncf2, Steap3, Txnip*, and *Gclc*.

Subsequently, SVM-RFE analysis results indicated that the 10 × CV error was minimized (0.0981) when the number of features was six. Thus, the model comprising six features (*Ncf2*, *Gclc*, *Steap3, Bach1, Hspb1,* and *Gch1*) was chosen as the optimal model (Fig. 4d).

Eventually, the genes chosen by the two models were combined to further screen diagnostic genes for sepsis-induced ALI, and three common genes (*Ncf2*, *Steap3*, and *Gclc*) were obtained (Fig. 4e). The AUCs of the ROC curves of the three genes in the training set were all greater than 0.7 (Fig. 4f), in which the diagnostic value of *Ncf2* and the combination model was confirmed in both of GSE165226 (Ncf2:AUC = 1, all:AUC = 1) and GSE168796 (Ncf2:AUC = 0.944, all:AUC = 0.972) datasets (Fig. 4g–h), indicating that the potential of these three genes as the diagnostic genes for sepsis-induced ALI.

### IPA and GSEA

**Fig. 5:**
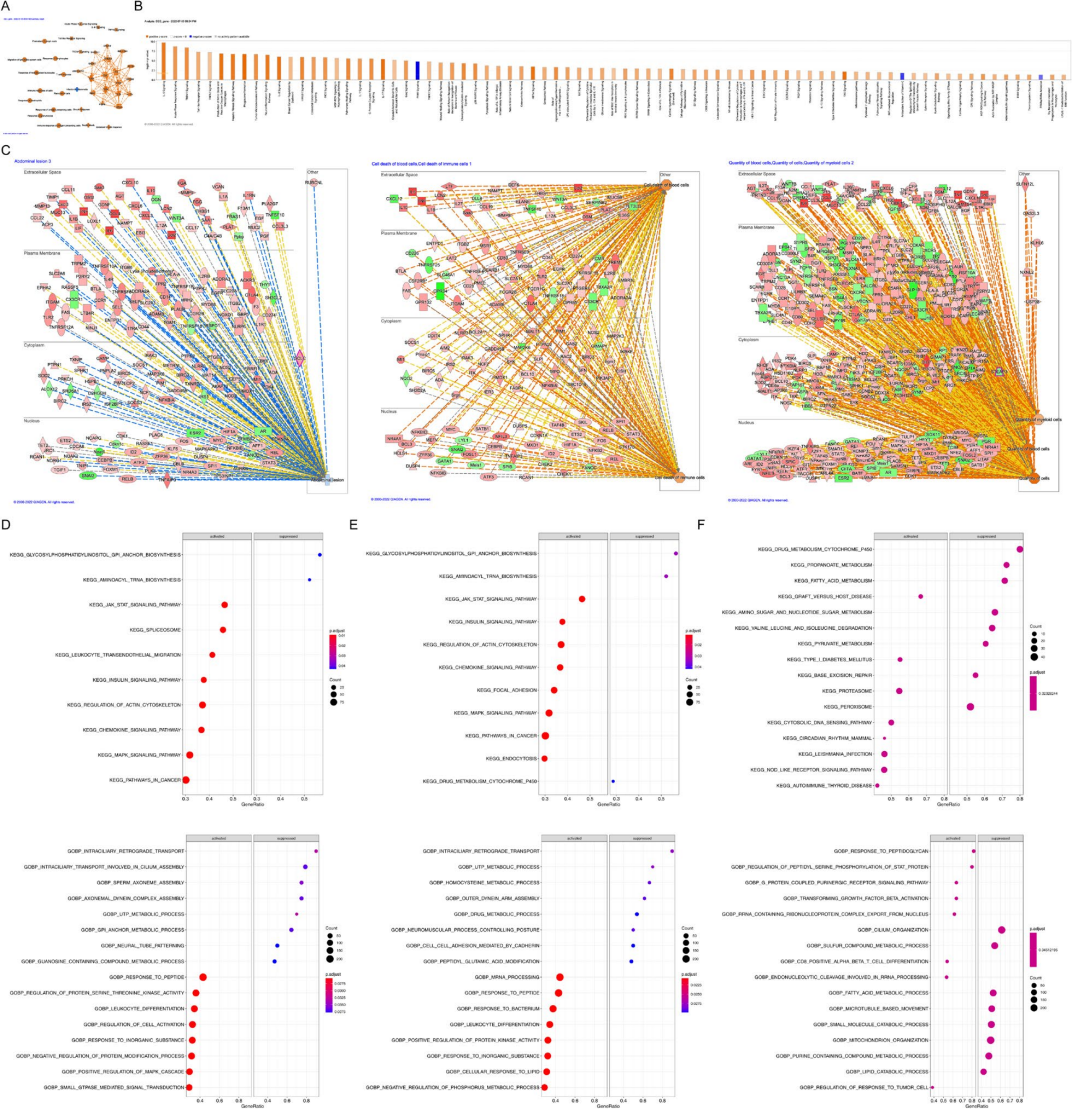
Ingenuity Pathway Analysis (IPA) and Gene Set Enrichment Analysis (GSEA) of key genes and FR-DEGs. (**a**) Summary graph of IPA. (**b**) Bar chart of enriched canonical pathways. (**c**) IPA functional pathway networks of *Ncf2*, *Steap3* and *Gclc*. **d**–**f** Single-gene GSEA dot plots of *Ncf2, Steap3*, and *Gclc*. Dot plots on the left are KEGG results and plots on the right are GO results. In the IPA summary graph, octagons represent cellular activity, hexagons represent signaling pathways, cross marks represent medical responses, and circles, ovals, and quadrilaterals represent genes. Orange represents activation, blue represents inhibition

The summary graph from IPA suggested that the DEGs were involved in disease effects such as septic shock, mammalian infection, and shock response. The major cellular activities involved were the immune cell activities, such as lymphatic system cell migration and T cell and lymphocyte responses. These symptoms and cellular activities are the main manifestations of sepsis-induced ALI (Fig. 5a). Specifically, 79 canonical pathways involved in pathways related to infection and immunity were screened and presented in the activation state (Fig. 5b). A total of six functional pathways were obtained which are active in disease, and two had Z scores greater than 2. *Ncf2* is involved in the cell death of blood and immune cells in combination with other DEGs. *Steap3* is involved in the quantity of myeloid cells, blood, and cells in general. *Gclc* is associated with abdominal lesions (Fig. 5c).

Furthermore, the GSEA results indicated that *Ncf2* was involved in activated BPs such as responses to peptides, regulation of protein serine threonine kinase activity, leukocyte differentiation, regulation of cell activation, and responses to inorganic substances. It was also involved in suppressed BPs such as neural tube patterning, UTP metabolic processes, regulation of cellular amino acid metabolic processes, and GPI anchor metabolic processes (Additional file 8: Table S4A, Fig. 5d). *Ncf2* was mainly involved in activated KEGG pathways including the chemokine, insulin, and JAK-STAT signaling pathways, and leukocyte transendothelial migration, as well as in the suppressed KEGG pathways of aminoacyl tRNA and glycosylphosphatidylinositol GPI anchor biosynthesis (Additional file 10: Table S4B).

Among the BPs involving *Steap3*, those in the activated state included mRNA processing, responses to peptides, bacterial responses, leukocyte differentiation, and responses to inorganic substances. Suppressed BPs included UTP metabolic processes, drug metabolism, and intraciliary retrograde transport. The KEGG pathways enriched by *Steap3* were activated as well, including the chemokine, insulin, and JAK-STAT signaling pathways and regulation of the actin cytoskeleton, Suppressed pathways included aminoacyl tRNA and glycosylphosphatidylinositol GPI anchor biosynthesis (Additional file 11: Table S4C, Additional file 12: S4D, Fig. 5e).

The enriched activated BPs related to *Gclc* included responses to peptidoglycan, transforming growth factor beta activation, G protein-coupled purinergic receptor signaling pathway, and regulation of STAT protein peptidyl serine phosphorylation. Suppressed BPs included fatty acid metabolism processes, small molecule catabolic processes, lipid catabolic processes, and cilium organization. In contrast to the other two key genes, the KEGG pathways most enriched by *Gclc* were mainly in the suppressed state, including peroxisome activity, pyruvate metabolism, fatty acid metabolism, propanoate metabolism, drug metabolism (cytochrome p450), and amino sugar and nucleotide sugar metabolism. Activated pathways included the proteasome and cytosolic DNA sensing pathways (Additional file 13: Table S4E, Additional file 14: S4F, Fig. 5f).

### Target miRNAs of the three key genes

**Fig. 6:**
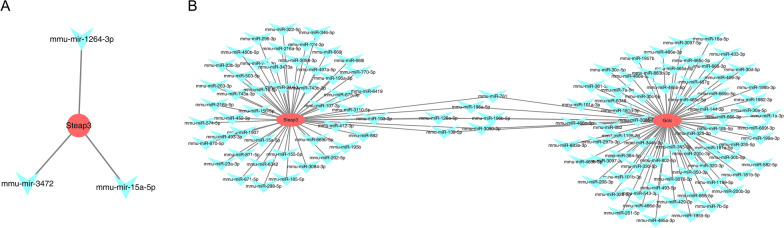
Target miRNAs of *Steap3* and *Gclc*. **a** An interaction network of *Steap3*. **b** An interacting network of *Steap3* and Gclc*.* The red dots in the figure represent key genes, and the blue diamonds represent interacting miRNAs

The prediction results from miRTarBase constructed a network consisting of one key gene (*Steap3*) and three miRNAs (mmu-mir-15a-5p, mmu-mir-3472, and mmu-mir-1264-3p) (Fig. 6a). Meanwhile, a network with two key genes (*Steap3* and *Gclc*) and 122 miRNAs was established from StarBase, of which six miRNAs were the common target miRNAs of *Steap3* and *Gclc*, namely mmu-miR-761, mmu-miR-196a-5p, mmu-miR-126a-5p, mmu-miR-196b-5p, mmu-miR-139-5p, and mmu-miR-3090-3p (Fig. 6b). Notably, mmu-miR-15a-5p was the target miRNA of *Steap3*, predicted by both databases.

### Expression validation of the three key genes

**Fig. 7:**
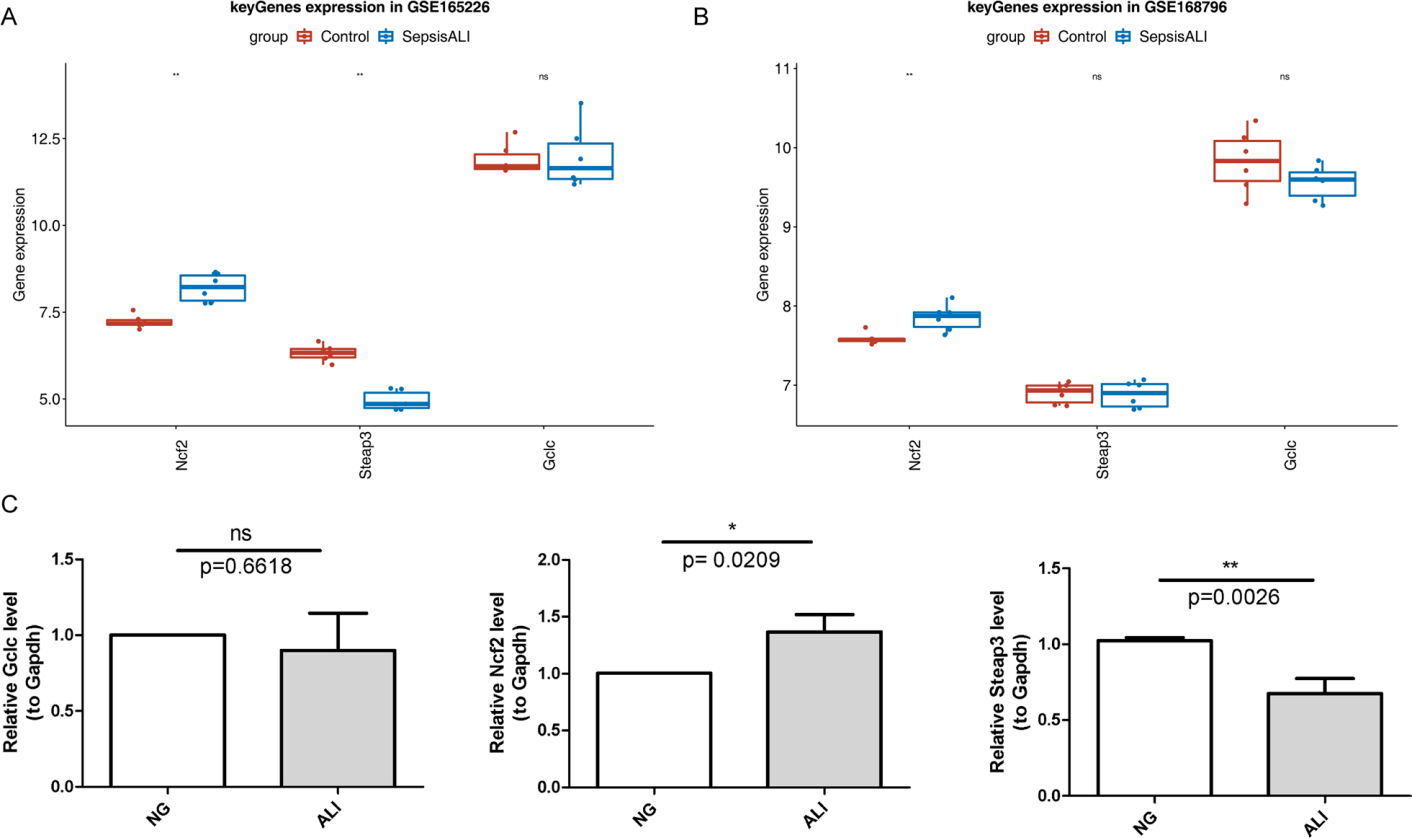
Validation of expression of *Ncf2*, *Steap3*, and *Gclc* using t-test. **a** Box plot of key gene expression between sepsis-acute lung injury (Sepsis-ALI) and control samples in GSE165226. **b** Box plot of key gene expression between sepsis-ALI and control samples in GSE168796. **c** qRT-PCR validation of *Ncf2* (Unpaired t test, *p* = 0.0209), *Steap3* (Unpaired t test, *p* = 0.0026), and *Gclc* (*p* = 0.6618). * *p* < 0.05, ** *p* < 0.01, *** *p* < 0.001

Validation results using GSE165226 showed that the expression of *Ncf2* was significantly higher in sepsis-induced ALI samples, whereas the expression of *Steap3* was higher in the control samples. There was no distinct difference in *Gclc* expression between the two groups (Fig. 7a). Additionally, in GSE168796, among the three key genes, distinct differences in expression between the two groups were only found for *Ncf2*, which was higher in sepsis-induced ALI samples (Fig. 7b). Therefore, the expressions of *Ncf2* and *Steap3* were consistent with the results of the sequencing analysis. There was no significant difference in *Gclc* expression between groups.

qRT-PCR validation showed that the expression of the three key genes was similar to the results from GSE165226, which found that *Ncf2* expression was higher in ALI samples, and *Steap3* was expressed at higher levels in control samples. There was no distinct difference in *Gclc* expression between the two groups (Additional file 7: Table 3, Fig. 7c).

## Discussion

Sepsis-induced ALI is the most common kind of ALI and currently lacks effective therapeutic measures [17]. Evidence has shown that ferroptosis plays an essential role in sepsis-induced ALI [18]. However, the regulatory mechanisms underlying ferroptosis in sepsis-induced ALI remain unclear. In the current study, according to the comprehensive analysis of transcriptomic data from a murine sepsis model, 24 FR-DEGs were first screened and analyzed in sepsis-induced ALI groups. Following the two machine learning methods, three FR-DEGs with diagnostic value in ALI were identified and explored through function enrichment analyses, IPA analysis as well as the construction of the genes-miRNAs network. Moreover, the differential expression of *Ncf2* and *Steap3* were verified in GSE165226, GSE168796 and qRT-PCR, which may provide a reference for the pathogenesis of sepsis-induced ALI from the perspective of bioinformatics.

The KEGG pathway enrichment results demonstrated that MAPK and NF-κB signaling, two significant inflammatory and immune pathways, were enriched by the 1843 DEGs. The MAPK pathway is associated with impaired tight junctions in lung microvascular endothelial cells during sepsis-induced ALI [19]. A previous study confirmed that suppression of sepsis-induced ALI may be achieved by blocking MAPK signaling via JNK and p38 phosphorylation in the lung tissue [20]. The NF-κB pathway is associated with sepsis-induced ALI. Butorphanol may facilitate the transition of macrophages from a pro-inflammatory to an anti-inflammatory phenotype in a mouse model of sepsis-induced ALI, which greatly reduces lung tissue damage via the NF-κB pathway [21]. Furthermore, research has established that lipid ROS produced by iron can activate MAPK and NF-κB [22, 23]. Therefore, the MAPK and NF-κB signaling pathways may be activated by the induction of ferroptosis and become involved in the pathogenesis of lung injury during sepsis.

GO analysis of 1843 DEGs showed that they are involved in immune cell migration and responses such as neutrophil infiltration. The early immune response in sepsis-induced ALI is dominated by neutrophils, and studies have shown that neutrophil extracellular traps (NETs), which have been proposed as an innate defense mechanism for pathogen clearance, were found abundantly in a sepsis-induced lung injury mouse model. Reduction of NETs may help reduce pulmonary injury [24]. Additionally, exosomal miR-30d-5p from polymorphonuclear neutrophils contributes to sepsis-related ALI by inducing M1 macrophage polarization and priming macrophage pyroptosis by activating NF-κB signaling [25], indicating that neutrophils are critically involved in sepsis-induced ALI.

Subsequently, two FR-DEGs associated with sepsis-induced ALI were further identified through transcriptome analysis, including *Ncf2* and *Steap3*. As the "activator" of the NADPH complex, Ncf2 is closely correlated with oxidase activity [26, 27]. The inflammatory response activates NADPH oxidase, which induces ROS formation, and ROS accumulation can lead to ferroptosis [28–30]. In addition, previous studies have shown that the suppression of Ncf2 leads to a decrease in NADPH oxidase activity and reduces the production of ROS [31, 32]. Furthermore, previous studies have shown that suppressing the activity of NAPDH oxidase could reduce ROS levels in lung tissues and protect the function of the pulmonary vascular barrier, alleviating sepsis-induced ALI [33, 34]. In the study, it was found that *Ncf2* expression was high in sepsis-induced ALI samples, confirming the relationship of *Ncf2* dysregulation and ALI progression. Xu et al. also found that Ncf2 was significantly higher in LPS-induced ALI rats than in the non-sepsis group [35], consistent with the trend of *Ncf2* expression that we obtained. Thus, it is conceivable to hypothesize that Ncf2 may participate in ferroptosis and the pathological process of sepsis-induced ALI by influencing the activity of NADPH oxidase and the production of ROS; however, this needs to be proven experimentally in the future.

Six-transmembrane epithelial antigen of prostate 3 (Steap3) was recently characterized as a ferrireductase that participates in cellular iron homeostasis by reducing Fe^3+^ to Fe^2+^ in endosomes [36, 37]. A direct cause of ferroptosis is the disruption of cellular iron metabolism. As a result of excessive *Steap3* expression, Fe^2+^ concentrations in the intracellular environment increase, which can lead to an imbalance in iron homeostasis, free radical generation, and lipid peroxidation, finally resulting in ferroptosis [38]. During hepatocyte ischemia–reperfusion injury, silencing of *Steap3* attenuates ferroptosis and reduces the levels of lipid-ROS and Fe^2+^ in H/R-treated cells; *Steap3* overexpression has the opposite effect [39]. In addition, in previous bioinformatics analyses of hepatocellular [40], basal cell (BCC), and squamous cell carcinomas [38], *Steap3* was identified as a novel diagnostic and prognostic gene associated with ferroptosis. We found that higher expression levels of *Steap3* in the control samples. Meanwhile, previous studies have shown that *Steap3* mRNA declined markedly in macrophages treated with LPS [41], which is similar to our results. Nevertheless, whether *Steap3* regulates sepsis-induced ALI through the ferroptosis pathway remains unclear.

IPA and GSEA were used to further analyze the potential pathways and biological functions of *Ncf2* and *Steap3*. Overall, the enrichment results for *Ncf2* and *Steap3* were similar. Our results showed that some inflammatory signal-relevant pathways were enriched by *Ncf2* and *Steap3*, such as the JAK-STAT, MAPK, and cytokine activation signaling pathways, which have been shown to be involved in ferroptosis [23, 42] and sepsis-induced lung injury [43, 44]. Sepsis is a disease characterized by inflammatory dysfunction, and some GO terms of inflammation, including leukocyte differentiation and bacterial responses, were identified in relation to Ncf2 and Steap3. In addition, IPA analysis revealed that *Ncf2* combined with other DEGs was involved in the cell death of blood and immune cells. Sepsis is caused by coagulation cascades and blood clotting by tissue factors released by pyroptotic macrophages after inflammasome activation [45]. In general, in the process of sepsis-induced ALI, *Ncf2* and *Steap3* mainly participate in disease pathways involved in infection, immunity, and inflammation.

Furthermore, in this study, diagnostic model construction and key gene screening for differential ferroptosis genes were achieved using LASSO and SVM-REF analyses. Regarding other diagnostic models of sepsis, Zhu et al. [46] identified two immune-related genes as potential diagnostic biomarkers for gram-negative sepsis via the same machine learning method. In the current study, it is the first to focus on the ferroptosis genes associated with sepsis-induced ALI prognosis. Notably, compared with the research of Zhu et al., our SVM-REF analysis is not only more reasonable in the choice of cross-validation multiples (10 × CV vs. 5 × CV) but also has lower error rates (0.0981 vs. 0.114). In addition, our AUCs of the key genes were better, indicating that the model possesses a strong ability to predict sepsis-induced ALI.

Furthermore, we predicted the interaction of miRNAs with key genes. MiR-15a-5p was predicted to be the target miRNA of *Steap3* by both databases. Previous studies have shown that downregulation of miR-15a-5p reduces apoptosis in sepsis-induced AKI [47]. In addition, in a mouse sepsis model, a miR-15a-5p inhibitor could reduce the activation of the NF-κB pathway by targeting TNIP2, significantly suppressing the secretion of inflammatory factors and the level of creatine and blood urea nitrogen, which could protect against sepsis [48]. In addition, there are still some potential associations between miR-139-5p and Gclc in acute lung injury. Studies have shown that inhibition of miR-139-5p can promote the phosphorylation of PI3K and AKT [49], while overexpression of Gclc can inhibit apoptosis of bone marrow mesenchymal stem cells through PI3K/AKT/Foxo1 pathway and alleviate inflammation caused by ALI [50], suggesting that the anti-apoptotic function of Gclc may be inhibited by miR-139-5p, while our study concluded that the KEGG pathway involved in Gclc genes was mainly in the inhibited state, which is inconsistent with our conclusion.

However, there are still some deficiencies in our research. First, our investigation only preliminarily predicted and validated diagnostic genes in sepsis-induced ALI, and further cellular and animal experiments are needed to confirm their exact regulatory roles on septic-induced ALI. Second, the experiments were conducted based on a septic mouse model, considering the species differences, more clinical samples need to be collected to validate the effectiveness of diagnostic genes for predicting and treating human sepsis ALI. Finally, there was no significant difference in *Gclc* expression between the groups, which may have been influenced by the sample size of the dataset or the sensitivity of the gene-expression assay platform. In the future, we will collect more clinical samples and focus on the expression and functional changes of diagnostic genes in ALI based on in vitro and in vivo experiments.

## Conclusions

Consequently, three key genes (*Ncf2*, *Steap3*, and *Gclc*) with good diagnostic efficiency in ALI were identified through transcriptome analyses, where *Ncf2* and *Steap3* were confirmed using two external validation sets and qRT-PCR. Inflammation- and immune-related signaling pathways, especially the JAK-STAT, MAPK, and cytokine activation may be potential pathways for the involvement of *Ncf2* and *Steap3* in ferroptosis and septic lung injury. The target miRNAs of Steap3 were considered as the potential regulators in ALI as well. The study is the first to report these results in the context of sepsis-induced ALI, and we are suggesting that thses key genes are closely associated with ferroptosis in sepsis-induced ALI and can differentiate sepsis from controls, which may be advantageous for the diagnosis and monitoring of ferroptosis.

## Supplementary Information

**Additional file 1.** Transcriptome Data after Quality Control.

**Additional file 2.** Clean Reads Mapped to GRCm39.

**Additional file 3.** Primers for qRT-PCR used in the current study.

**Additional file 4.** The statistical results of three key genes in qRT-PCR.

**Additional file 5.** The gene expression of each sample.

**Additional file 6.** Table of biological processes of Ncf2.

**Additional file 7.** Table of KEGG pathways of Ncf2.

**Additional file 8.** Table of biological processes of Steap3

**Additional file 9.** Table of KEGG pathways of Steap3.

**Additional file 10.** Table of biological processes of Gclc.

**Additional file 11.** Table of KEGG pathways of Gclc.

**Additional file 12.** Quality scores across all bases.

**Additional file 13.** Sequence content across all bases.

**Additional file 14.** Transcriptome sequencing sample information.

## Data Availability

Data analyzed in this manuscript are publicly available from the Sequence Read Archive(https://www.ncbi.nlm.nih.gov/sra/PRJNA899441).

## Abbreviations

ALI: Acute lung injury
BCC: Basal cell
BP: Biological processes
CLP: Cecal ligation and puncture
DEG: Differentially expressed gene
FRG: Ferroptosis-related gene
GEO: Gene expression omnibus
GSEA: Gene set enrichment analysis
HE: Hematoxylin and eosin
IPA: Ingenuity pathway analysis
MDA: Malondialdehyde
MF: Molecular function
NET: Neutrophil extracellular trap
PCA: Principal component analysis
PPI: Protein–protein interaction
qRT-PCR: Quantitative real-time polymerase chain reaction
ROS: Reactive oxygen species
SVM-RFE: Support vector machine-recursive feature elimination

## References

[1] Verdonk F, Blet A, Mebazaa A (2017). The new sepsis definition: limitations and contribution to research and diagnosis of sepsis. Curr Opin Anaesthesiol.

[2] Iscimen R (2008). Risk factors for the development of acute lung injury in patients with septic shock: an observational cohort study. Crit Care Med.

[3] Mokra D, Kosutova P (2015). Biomarkers in acute lung injury. Respir Physiol Neurobiol.

[4] Kumar V, Chhibber S (2011). Acute lung inflammation in Klebsiella pneumoniae B5055-induced pneumonia and sepsis in BALB/c mice: a comparative study. Inflammation.

[5] Stapleton RD (2005). Causes and timing of death in patients with ARDS. Chest.

[6] Dixon SJ (2012). Ferroptosis: an iron-dependent form of nonapoptotic cell death. Cell.

[7] Li N (2020). Ferritinophagy-mediated ferroptosis is involved in sepsis-induced cardiac injury. Free Radic Biol Med.

[8] Li J (2021). Panaxydol attenuates ferroptosis against LPS-induced acute lung injury in mice by Keap1-Nrf2/HO-1 pathway. J Transl Med.

[9] Liu P (2020). Ferrostatin-1 alleviates lipopolysaccharide-induced acute lung injury via inhibiting ferroptosis. Cell Mol Biol Lett.

[10] Mishima E (2020). Drugs repurposed as antiferroptosis agents suppress organ damage, including AKI, by functioning as lipid peroxyl radical scavengers. J Am Soc Nephrol.

[11] He R (2022). Itaconate inhibits ferroptosis of macrophage via Nrf2 pathways against sepsis-induced acute lung injury. Cell Death Discov.

[12] Rittirsch D (2009). Immunodesign of experimental sepsis by cecal ligation and puncture. Nat Protoc.

[13] Zhang W (2021). Restoring microRNA-499-5p protects sepsis-induced lung injury mice via targeting Sox6. Nanoscale Res Lett.

[14] Kanehisa M, Goto S (2000). KEGG: kyoto encyclopedia of genes and genomes. Nucleic Acids Res.

[15] Kanehisa M (2019). Toward understanding the origin and evolution of cellular organisms. Protein Sci.

[16] Kanehisa M (2023). KEGG for taxonomy-based analysis of pathways and genomes. Nucleic Acids Res.

[17] Cao X (2019). MiR-145 negatively regulates TGFBR2 signaling responsible for sepsis-induced acute lung injury. Biomed Pharmacother.

[18] Yin X (2021). Ferroptosis, a new insight into acute lung injury. Front Pharmacol.

[19] Liu Y (2019). Unfractionated heparin alleviates sepsis-induced acute lung injury by protecting tight junctions. J Surg Res.

[20] Fang W (2017). Modulation of mitogen-activated protein kinase attenuates sepsis-induced acute lung injury in acute respiratory distress syndrome rats. Mol Med Rep.

[21] Luan G (2021). Butorphanol promotes macrophage phenotypic transition to inhibit inflammatory lung injury via κ receptors. Front Immunol.

[22] Yan N (2021). Dimethyl fumarate improves cognitive deficits in chronic cerebral hypoperfusion rats by alleviating inflammation, oxidative stress, and ferroptosis via NRF2/ARE/NF-κB signal pathway. Int Immunopharmacol.

[23] Nakamura T, Naguro I, Ichijo H (2019). Iron homeostasis and iron-regulated ROS in cell death, senescence and human diseases. Biochim Biophys Acta Gen Subj.

[24] Lefrançais E (2018). Maladaptive role of neutrophil extracellular traps in pathogen-induced lung injury. JCI Insight.

[25] Jiao Y (2021). Exosomal miR-30d-5p of neutrophils induces M1 macrophage polarization and primes macrophage pyroptosis in sepsis-related acute lung injury. Crit Care.

[26] Italiano D (2012). Identification of NCF2/p67phox as a novel p53 target gene. Cell Cycle.

[27] El-Benna J, Dang PM (2021). Starting-NOX2-Up: Rac unrolls p67(phox). J Leukoc Biol.

[28] Chiriaco M (2016). Chronic granulomatous disease: clinical, molecular, and therapeutic aspects. Pediatr Allergy Immunol.

[29] Martner A, Aydin E, Hellstrand K (2019). NOX2 in autoimmunity, tumor growth and metastasis. J Pathol.

[30] Tang D (2021). Ferroptosis: molecular mechanisms and health implications. Cell Res.

[31] Ammons MC (2007). Binding of pleomorphic adenoma gene-like 2 to the tumor necrosis factor (TNF)-alpha-responsive region of the NCF2 promoter regulates p67(phox) expression and NADPH oxidase activity. J Biol Chem.

[32] Abais-Battad JM (2020). NOX2-derived reactive oxygen species in immune cells exacerbates salt-sensitive hypertension. Free Radic Biol Med.

[33] Fisher AB (2019). A peptide inhibitor of NADPH oxidase (NOX2) activation markedly decreases mouse lung injury and mortality following administration of lipopolysaccharide (LPS). Int J Mol Sci.

[34] Jiang J (2020). Targeting NOX4 alleviates sepsis-induced acute lung injury via attenuation of redox-sensitive activation of CaMKII/ERK1/2/MLCK and endothelial cell barrier dysfunction. Redox Biol.

[35] Xu L (2014). Protective effects of apocynin nitrone on acute lung injury induced by lipopolysaccharide in rats. Int Immunopharmacol.

[36] Ohgami RS (2005). Identification of a ferrireductase required for efficient transferrin-dependent iron uptake in erythroid cells. Nat Genet.

[37] Muckenthaler MU (2017). A red carpet for iron metabolism. Cell.

[38] Wang J (2022). Ferroptosis-related local immune cytolytic activity in tumor microenvironment of basal cell and squamous cell carcinoma. Aging (Albany NY).

[39] Wu L (2022). miR-124-3p delivered by exosomes from heme oxygenase-1 modified bone marrow mesenchymal stem cells inhibits ferroptosis to attenuate ischemia-reperfusion injury in steatotic grafts. J Nanobiotechnology.

[40] Yan Y (2021). Downregulated ferroptosis-related gene *STEAP3* as a novel diagnostic and prognostic target for hepatocellular carcinoma and its roles in immune regulation. Front Cell Dev Biol.

[41] Zhang F (2012). Metalloreductase *Steap3* coordinates the regulation of iron homeostasis and inflammatory responses. Haematologica.

[42] Yu X (2022). IFNγ enhances ferroptosis by increasing JAK-STAT pathway activation to suppress SLCA711 expression in adrenocortical carcinoma. Oncol Rep.

[43] Zhao J (2016). Protective effect of suppressing STAT3 activity in LPS-induced acute lung injury. Am J Physiol Lung Cell Mol Physiol.

[44] Roquilly A (2017). Local modulation of antigen-presenting cell development after resolution of pneumonia induces long-term susceptibility to secondary infections. Immunity.

[45] Wu C (2019). Inflammasome activation triggers blood clotting and host death through pyroptosis. Immunity.

[46] Zhu D, Zhu K, Guo S (2022). Identification of key genes related to immune cells in patients with gram-negative sepsis based on weighted gene co-expression network analysis. Ann Transl Med.

[47] Xu G (2019). The miR-15a-5p-XIST-CUL3 regulatory axis is important for sepsis-induced acute kidney injury. Ren Fail.

[48] Lou Y, Huang Z (2020). microRNA-15a-5p participates in sepsis by regulating the inflammatory response of macrophages and targeting TNIP2. Exp Ther Med.

[49] Mo FF (2017). Jiang Tang, Xiao Ke granule play an anti-diabetic role in diabetic mice pancreatic tissue by regulating the mRNAs and MicroRNAs associated with PI3K-Akt signaling pathway. Front Pharmacol.

[50] Zhang Z (2022). Gclc overexpression inhibits apoptosis of bone marrow mesenchymal stem cells through the PI3K/AKT/Foxo1 pathway to alleviate inflammation in acute lung injury. Int Immunopharmacol.

