# Supplementary material for: Identification of ferroptosis-related genes in male mice with sepsis-induced acute lung injury based on transcriptome sequencing
Source: BMC Pulm Med. 2023 Apr 20;23:133. doi: 10.1186/s12890-023-02361-3 (PMC10116744; doi:10.1186/s12890-023-02361-3)
Supplement: Supplementary file 1 — Additional file 1. Transcriptome Data after Quality Control. [file 12890_2023_2361_MOESM1_ESM.docx]

**Supplementary Table 1B: Transcriptome Data after Quality Control**

| **Sample ID** | **LENGTH** | **READS** | **QC30%** | **DC%** |
| --- | --- | --- | --- | --- |
| GY9A1 | 36-150 | 19939849*2 | 100% | 52 |
| GY9A2 | 36-150 | 36139567*2 | 100% | 48 |
| GY9A3 | 36-150 | 34607970*2 | 100% | 48 |
| GY9A4 | 36-150 | 35098065*2 | 100% | 49 |
| GY9A5 | 36-150 | 32957568*2 | 100% | 49 |
| GY9A6 | 36-150 | 38214345*2 | 100% | 49 |
| GY9A7 | 36-150 | 29579247*2 | 100% | 48 |
| GY9A8 | 36-150 | 27260291*2 | 100% | 49 |
| GY9A9 | 36-150 | 25089138*2 | 100% | 49 |
| GY9A10 | 36-150 | 41585637*2 | 100% | 48 |
| GY9B1 | 36-150 | 38885821*2 | 100% | 48 |
| GY9B2 | 36-150 | 35664728*2 | 100% | 49 |
| GY9B3 | 36-150 | 36819651*2 | 100% | 49 |
| GY9B4 | 36-150 | 36888020*2 | 100% | 49 |
| GY9B5 | 36-150 | 27669360*2 | 100% | 49 |
| GY9B6 | 36-150 | 18732938*2 | 100% | 48 |
| GY9B7 | 36-150 | 27024559*2 | 100% | 48 |
| GY9B8 | 36-150 | 41871029*2 | 100% | 49 |
| GY9B9 | 36-150 | 33946774*2 | 100% | 48 |
| GY9B10 | 36-150 | 36069109*2 | 100% | 48 |
