# Supplementary material for: Identification of ferroptosis-related genes in male mice with sepsis-induced acute lung injury based on transcriptome sequencing
Source: BMC Pulm Med. 2023 Apr 20;23:133. doi: 10.1186/s12890-023-02361-3 (PMC10116744; doi:10.1186/s12890-023-02361-3)
Supplement: Supplementary file 2 — Additional file 2. Clean Reads Mapped to GRCm39. [file 12890_2023_2361_MOESM2_ESM.docx]

**Supplementary Table 1C: Clean Reads Mapped to GRCm39**

| **Sample ID** | **TOTAL_READS (QC-PASSED READS + QC-FAILED READS)** | **TOTAL_MAPPED** | **PAIRED IN SEQUENCING** | **PROPERLY PAIRED** |
| --- | --- | --- | --- | --- |
| GY9A1 | 43457017 | 42091718(96.86%) | 39879698 | 36991630(92.76%) |
| GY9A2 | 79822894 | 77820413(97.49%) | 72279134 | 67793728(93.79%) |
| GY9A3 | 76042797 | 73676957(96.89%) | 69215940 | 63941514(92.38%) |
| GY9A4 | 79268578 | 76439061(96.43%) | 70196130 | 64073252(91.28%) |
| GY9A5 | 70751378 | 68375863(96.64%) | 65915136 | 60624764(91.97%) |
| GY9A6 | 83369328 | 80896339(97.03%) | 76428690 | 70912784(92.78%) |
| GY9A7 | 69451548 | 67421235(97.08%) | 59158494 | 55267044(93.4%) |
| GY9A8 | 58981371 | 57726091(97.87%) | 54520582 | 51574800(94.60%) |
| GY9A9 | 55129766 | 53853547(97.69%) | 50178276 | 47108578(93.88%) |
| GY9A10 | 90041913 | 86639572(96.22%) | 83171274 | 75401428(90.66%) |
| GY9B1 | 84920891 | 82014951(96.58%) | 77771642 | 71137672(91.47%) |
| GY9B2 | 78144855 | 76050194(97.32%) | 71329456 | 66571498(93.33%) |
| GY9B3 | 80284224 | 77087047(96.02%) | 73639302 | 66315680(90.05%) |
| GY9B4 | 81199914 | 78152465(96.25%) | 73776040 | 67438266(91.41%) |
| GY9B5 | 63796078 | 61387690(96.22%) | 55767624 | 50277486(90.16%) |
| GY9B6 | 60149904 | 58293393(96.91%) | 55338720 | 51240142(92.59%) |
| GY9B7 | 60328229 | 58451109(96.89%) | 54049118 | 49964876(92.44%) |
| GY9B8 | 92287006 | 89384920(96.86%) | 83742058 | 77298128(92.31%) |
| GY9B9 | 76137978 | 73817294(96.95%) | 67893548 | 62746128(92.42%) |
| GY9B10 | 79797766 | 75887048(95.10%) | 67893548 | 62746128(92.42%) |
