# Supplementary material for: Identification of ferroptosis-related genes in male mice with sepsis-induced acute lung injury based on transcriptome sequencing
Source: BMC Pulm Med. 2023 Apr 20;23:133. doi: 10.1186/s12890-023-02361-3 (PMC10116744; doi:10.1186/s12890-023-02361-3)
Supplement: Supplementary file 3 — Additional file 3. Primers for qRT-PCR used in the current study. [file 12890_2023_2361_MOESM3_ESM.docx]

**Supplementary** **Table 2: Primers for qRT-PCR used in the current study**

| Primer | Sequence | |
| --- | --- | --- |
| Ncf2 F | CACCAGGTCACAAGCAAAAAGA | |
| Ncf2 R | CACACCACAGAGTCAGGCAGTA | |
| Steap3 F | CCTCCTTAGCTCAAGTGACTTTC |  |
| Steap3 R | TGCACAGTGAGGAATAGTGCT |  |
| Gclc F | GGCTTCTCAGCCAGACCATA |  |
| Gclc R | ACTCCCCAGCGACAATCAAT |  |
| internal reference H-GAPDH F | CCTTCCGTGTTCCTACCCC |  |
| internal reference H-GAPDH R | GCCCAAGATGCCCTTCAGT |  |
