# Supplementary material for: Identification of ferroptosis-related genes in male mice with sepsis-induced acute lung injury based on transcriptome sequencing
Source: BMC Pulm Med. 2023 Apr 20;23:133. doi: 10.1186/s12890-023-02361-3 (PMC10116744; doi:10.1186/s12890-023-02361-3)
Supplement: Supplementary file 4 — Additional file 4. The statistical results of three key genes in qRT-PCR. [file 12890_2023_2361_MOESM4_ESM.docx]

**Supplementary Table 3** The statistical results of three key genes in qRT-PCR .

|  | NG | ALI | t, df value | P value |
| --- | --- | --- | --- | --- |
| Ncf2 | 1.0047±0.0039 | 1.3682±0.4246 | t=2.579 df=15 | 0.0209 |
| Steap3 | 1.0231±0.0648 | 0.6741±0.2834 | t=3.604 df=15 | 0.0026 |
| Gclc | 1.0024±0.0017 | 0.8990±0.6975 | t=0.4462 df=15 | 0.6618 |

NG: control mice; ALI: sepsis-induced acute lung injury mice
