# Supplementary figures and images for: Identification of ferroptosis-related genes in male mice with sepsis-induced acute lung injury based on transcriptome sequencing
Source: BMC Pulm Med. 2023 Apr 20;23:133. doi: 10.1186/s12890-023-02361-3 (PMC10116744; doi:10.1186/s12890-023-02361-3)

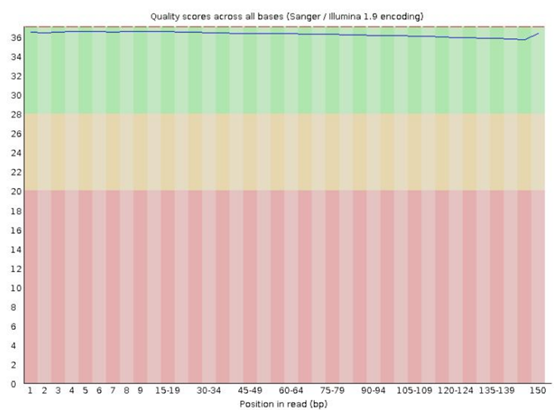

Supplement: Supplementary file 12 — Additional file 12. Quality scores across all bases. [file 12890_2023_2361_MOESM12_ESM.tif]

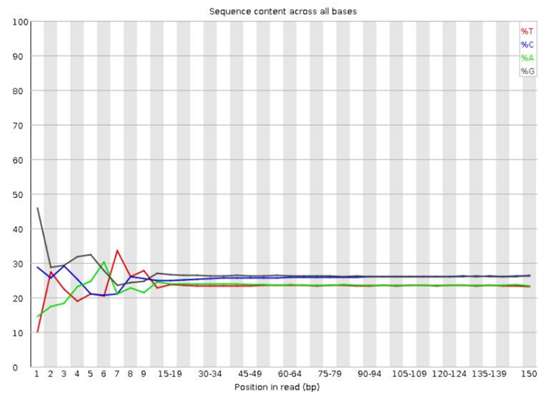

Supplement: Supplementary file 13 — Additional file 13. Sequence content across all bases. [file 12890_2023_2361_MOESM13_ESM.tif]
