# Supplementary material for: Identification of ferroptosis-related genes in male mice with sepsis-induced acute lung injury based on transcriptome sequencing
Source: BMC Pulm Med. 2023 Apr 20;23:133. doi: 10.1186/s12890-023-02361-3 (PMC10116744; doi:10.1186/s12890-023-02361-3)
Supplement: Supplementary file 14 — Additional file 14. Transcriptome sequencing sample information. [file 12890_2023_2361_MOESM14_ESM.docx]

Supplementary Table 1: Transcriptome sequencing sample information

| Sample ID | Control | Sepsis_ALI |
| --- | --- | --- |
| GY9A1 | YES |  |
| GY9A2 | YES |  |
| GY9A3 | YES |  |
| GY9A4 | YES |  |
| GY9A5 | YES |  |
| GY9A6 | YES |  |
| GY9A7 | YES |  |
| GY9A8 | YES |  |
| GY9A9 | YES |  |
| GY9A10 | YES |  |
| GY9B1 |  | YES |
| GY9B2 |  | YES |
| GY9B3 |  | YES |
| GY9B4 |  | YES |
| GY9B5 |  | YES |
| GY9B6 |  | YES |
| GY9B7 |  | YES |
| GY9B8 |  | YES |
| GY9B9 |  | YES |
| GY9B10 |  | YES |
